# Masculinities and condom use patterns among young rural South Africa men: a cross-sectional baseline survey

**DOI:** 10.1186/1471-2458-12-462

**Published:** 2012-06-20

**Authors:** N Jama Shai, R Jewkes, M Nduna, K Dunkle

**Affiliations:** 1 Gender & Health Research Unit, Medical Research Council (MRC), Pretoria, South Africa; 2 School of Public Health, University of the Witwatersrand, Johannesburg, South Africa; 3 Department of Psychology, University of the Witwatersrand, Johannesburg, South Africa; 4 Behavioral Sciences and Health Education, Emory University, Atlanta, GA, USA

**Keywords:** Condom use, Masculinities, Sexual behaviour, Young men, South Africa

## Abstract

**Background:**

Notions of ideal manhood in South Africa are potentially prescriptive of male sexuality thus accounting for the behaviors which may lead to men being at greater HIV risk. We tested the hypothesis that gender and relationship constructs are associated with condom use among young men living in rural South Africa.

**Methods:**

1219 men aged 15–26 years completed a cross-sectional baseline survey from an IsiXhosa questionnaire asking about sexual behaviour and relationships. Univariate and bivariate analyses described condom use patterns and explanatory variables, and multinomial regression modeling assessed the factors associated with inconsistent versus consistent and non-condom use.

**Results:**

47.7% of men never used condoms, when 36.9% were inconsistent and 15.4% were consistent with any partner in the past year. Condom use patterns differed in association with gender relations attitudes: never users were significantly more conservative than inconsistent or consistent users. Three gender positions emerged indicating that inconsistent users were most physically/sexually violent and sexually risky; never users had more conservative gender attitudes but were less violent and sexually risky; and consistent users were less conservative, less violent and sexually risky with notably fewer sexual partners than inconsistent users.

**Conclusions:**

The confluence of conservative gender attitudes, perpetration of violence against women and sexual risk taking distinguished inconsistent condom users as the most risky compared to never condom users, and rendered inconsistent use one of the basic negative attributes of dominant masculinities in the Eastern Cape, South Africa. This finding is important for the design of HIV prevention and gender equity interventions and emphasizes the need for a wider roll-out of interventions that promote progressive and healthy masculine practices in the country.

## Background

Young men are vulnerable for contracting HIV infection due to a tendency to engage in unprotected sexual intercourse [1]. A national youth study found that two-thirds of youth aged 15–24 years had used a condom [2] and 33.5% of men reported consistent use with the most recent partner, compared with 35.1% inconsistent and 31.3% never use [3]. Studies undertaken to understand patterns of condom use have often focused on women’s experiences [4], but understanding men’s experiences is also important for informing HIV risk reduction and developing strategies for engaging men and boys in the fight against HIV. Interventions that seek to promote condom use among women often fail to do so because men control condom use[4]. Thus male power in relationships is pertinent in determining safer sexual behaviour and significantly influences HIV risk. South African research on HIV prevention indicates that gender inequity in relationships greatly limits women’s safer sexual practices [5-7] and greater male power in sexual relationships accounts for much of the spread of HIV amongst women [7-10]. Understanding what factors influence men’s ideas and practices related to condoms is valuable for explaining why men do not use condoms.

Non-condom use, as well as inconsistent use, among men cannot be attributed to a single factor. A complex web of factors influence why some men have never engaged in protected sex and why, among those who have, condom use is inconsistent. Following the principles of the ecological model[11], condom use is influenced by dynamics operating on multiple levels, that is, individual factors, the relationship dyad, family, peers and community/societal contexts within which individuals live. At the individual level lower perceptions of personal HIV risk [12] have been associated with non- and inconsistent condom use. At the dyad level, condoms may be seen as interruptive agents against trust and intimacy and sexual pleasure experienced [12,13]. Studies show contradictions in condom use depending on the status of a relationship: while it can be uncommon with main partners, there are instances where use is also inconsistent with casual partners [14] albeit the perception of its appropriateness in casual rather than main sexual relationships [15]. At a community/societal level, men who share conservative ideas about gender, such as notions and practices that uphold views about male superior status over females, anti-femininity and male hypersexuality, seldom use condoms [16]. Yet, consistent condom use is possible when there is high gender equity and less conflict in relationships[7,17]. Since South African research indicates that many young men have used condoms at least once in their lives [18], the study seeks to explore why consistency of use is not the norm.

Conservative gender norms, roles and attitudes [4,16], perpetration of physical or sexual violence against a female intimate partner and other women [19-22], transactional sex, alcohol abuse [23], and multiple concurrent partners [24,25] are significant markers of HIV risk. Risky sexual practices of men are also strongly correlated with less gender equitable attitudes [26,27]. These ideas about gender greatly influence the formation of masculine gender identities and their role in legitimizing and promoting male ascendancy over other men and women in society, including their partners [28]. Connell [29] also refers to the concept of hegemonic masculinity as representing a configuration of beliefs and practices constituting an ‘ideal’ manhood. Hegemony signifies the extent to which one form of masculinity dominates over other (alternative) masculinities, and exists with the simultaneous consent and participation of other non-hegemonic forms. Connell maintains that although not universal, hegemonic masculinity evolves over time, adapting aspects of other masculinities to reinforce its dominance over them, and performing an array of both potentially constructive and destructive traits. On their own, ideals of manhood are not all harmful, however in the era of promoting HIV prevention and gender equity, certain elements of male ideology are a cause for concern, for instance, male toughness and virility are offset against expectations that men fulfill the protector role and can translate into risky sexual and anti-social practices. Moreover, hegemony is not regulated by violence, yet violence can be used in the assertion of the notions of being a man in certain settings [28]. In the South African context, authors have argued that male toughness, perpetration of violence, acquisition of many sexual partners, and even non- or inconsistent condom use flow from hegemonic masculinity [30], with its demonstrations of male control over female partners and heterosexual prowess. Men who aspire to embrace hegemonic masculinity are more likely to support and engage in these practices, and form an important group on which to focus reducing HIV risk reduction efforts. It is not always clear how condom use is influenced by men’s gender attitudes and behaviours, thus it is appropriate to investigate how ideals of masculinity may influence young men’s condom use behaviour, and in turn, to reflect on whether changes in ideals of masculinity have potential for reducing HIV risk.

In this paper we examine the hypothesis that the nature of male gender identity influences patterns of condom use amongst rural young men living in the Eastern Cape, South Africa. We will examine the associations between aspects of gender and relationships and violence and risky sexual practices, and three categories of condom use, that is, inconsistent condom use in comparison with consistent and non-condom use.

## Methods

Cross-sectional data from 1219 baseline interviews conducted with male volunteers in a community randomised controlled trial (RCT) to evaluate the Stepping Stones behavioural HIV prevention intervention between 2003–2006 was obtained [31]. The trial was implemented in rural and peri-urban communities within a 1.5 h’s drive radius from the central town of Mthatha in the Eastern Cape, South Africa, with men and women. The area comprises of a few small towns and population dispersed across many rural villages. 70 villages were selected to form clusters from which two single sex groups of 20 members could be recruited. These villages were about 10 or more kilometers apart, many had a clinic nearby but most had schools within them. Due to inadequate access to out of school youth, recruitment was mostly conducted in schools. Further details on the RCT are described [32,33]. Male participants were Black IsiXhosa-speaking youth aged 15–26 years (a majority of whom were under the age of 20 years), a group that is relatively marginalized in relation to employment, and often overlooked in relation to access to sexual and reproductive health services, partly due to traditional ideology about men in the area. Their backgrounds are marked by starkly high levels of unemployment and lower literacy amongst adult family members and parents/guardians who depend on subsistence farming, or low-wages if they are working or government social grants. This paper presents an analysis of the data from 1219 sexually active men who reported having had a main or a casual partner in the 12 months prior to the baseline interview.

### Measurement tools

Interviews were conducted by male interviewers of similar age using a structured isiXhosa questionnaire. The questionnaire collected information regarding socio-demographic factors, gender attitudes, sexual experiences, details on the most recent relationship and perpetration of gender based violence.

### Condom use outcome measures

The outcome measure is condom use with any partner in the year prior to the baseline interviews and has been classified into three categories: (1) Inconsistent condom use is defined as instances where men reported having used condoms sometimes or often, but not always, or had used condoms but not at last sex, or used condoms but not always correctly (i.e. experiences of a condom breaking, slipping off, being taken off during intercourse or being put on late); (2) No condom use is defined as instances where men reported never having used a condom with any partner in the past year; (3) Consistent condom use is referred to occasions where men reported always using it correctly and at last sex (see Table 1).

**Table 1:** Summary of data socio-demographic, risky sexual and violent practices and condom use patterns among 1219 young men aged 15–26 years

	**Frequency (%)**	**Mean (95% Confidence Interval)**
**Socio-demographic factors**		***Mean (95% CI)***
Age in years	-	19.23 (19.06; 19.40)
Socio-economic status score	-	0.01 (−0.14; 0.15)
Ever earning money	673 (55.3%)	55.3 (51.4; 59.1)
Educated (up to 10 or more years)	550 (45.2%)	45.2 (39.8; 50,5)
Sexual activity practices		
3 or more partners in past year	635 (52.1%)	52.1 (49.3:55.0)
Condom use patterns		
Ever used a condom	814 (66.78%)	66.8 (62.9; 70.6)
Condoms at last sex	573 (47.01%)	.47.0 (43.3: 50.7)
Correct condom use at last sex (n = 573)	509 (88.83%)	-
Condom use with any partner in the past year		67.8 (62.5; 73.0)
Consistent (always and correctly at last sex)	188 (15.42%)	-
Inconsistent (often/sometimes/incorrectly at last sex)	450 (36.92%)	-
Never	581 (47.66%)	-
Gender Based Violence practices		
Perpetrated physical or sexual abuse of a partner in the past year	317 (26.1%)	26.1 (23.3; 28.9)
Ever perpetrated rape of a non-partner	222 (18.2%)	18.2 (15.8; 20.6)
Violence against women patterns:		
No violence	810 (67.0%)	-
Physical violence only	286 (23.7%)	-
Sexual violence only	47 (3.9%)	-
Physical and Sexual Violence	66 (5.5%)	

### Explanatory variables

The explanatory variables are shown in Table 2. These variables are defined in Jewkes et al. (2006) [32]. The socio-economic status (SES) scale groups 5 questions related to household goods ownership (TV, radio, and car), frequency of hunger, frequency of having meat, and perceived difficulty accessing a modest sum for a medical emergency (R100 or £8 or $14), with a mean of 0.01 and a range of −2.92 to 3.52. Tertiles of the scale show that 53.1% of participants scored below 0.49, that is, very low SES. Participants were asked about having done something to earn money. The gender attitudes and partner control scale (total of 13 items with lowest score = 13 and maximum score being 47) have been combined and adjusted to maximize internal consistency (Cronbach Alpha = 0.69). Tertiles of the scale were derived with low gender equity, middle and high gender equity and were analysed. Participants answered questions on their sexual experiences, including having ever had sex, time of last sex and the number of sexual partners in the past year. Questions about men’s perpetration of physical and sexual violence towards a female sexual partner were asked using a slightly adapted version of the WHO violence against women instrument [34], which was designed for use in developing countries. A four-level composite variable of violence against women was derived with (0) no violence (1) physical violence only (2) sexual violence only (3) physical or sexual violence. Following Jewkes et al. [26], rape of non-intimate partners was assessed by questions: ‘Was there a time when you made a woman or girl, other than your girlfriend at the time, have sex with you when she did not want to?’ and ‘Was there a time when you made a woman or girl, other than your girlfriend at the time, have sex with you when she was too drunk to say whether she wanted it?’ Two gang rape questions were ‘Have you ever done streamlining?’ and ‘Was there ever an occasion when you and other men had sex with a woman against her will or when she was too drunk to stop you?’ A man was considered to have ever raped a non-partner if he responded affirmatively to any individual or group perpetration question.

**Table 2:** Associations between condom-use categories and socio-demographic, attitudes, sexual behavioural and relationship characteristics of men, n = 1219

**Variables**	**Never: n = 581**		**Inconsistent: n = 450**		**Consistent: n = 188**	
	**n (%)/mean**	**95% CI**	**n (%)/mean**	**95% CI**	**n (%)/mean**	**95% CI**
Socio-demographic factors						
Age	19.08	18.9, 19.29	19.39	19.17, 19.60	19.3	18.96,19.64
Socio-economic status score	−0.21	−0.36, −0.06	0.20	0.02, 0.38	0.22	−0.05, 0.48
Ever done something to earn money	283 (42.1%)	44.0, 53.6	269 (59.8%)	55.3, 64.2	121 (64.4%)	56.5, 72.2
Attitudes						
Having gender relations attitudes & relationship control scale (high = liberal)	−0.13	−0.26, −0.00	0.07	−0.03, 0.17	0.23	0.03, 0.43
Sexual practices						
Had 3 sexual partners or more in the past year	193 (33.3%)	29.2, 37.3	369 (82.0%)	78.3, 85.7	73 (38.8%)	31.8, 45.9
Gender-based Violence						
Ever perpetrated physical or sexual intimate partner violence (IPV)	164 (28.6%)	24.6, 32,5	183 (40.8%)	35.2, 46.2	49 (26.3%)	19.3, 33.4
Perpetrating physical or sexual intimate partner violence (IPV) in the past year	135 (23.4%)	19.7, 27.1	147 (32.7%)	27.9, 37.4	35 (18.7%)	12.7, 24.8
Ever perpetrated rape of non-partner	66 (11.4%)	8.29, 14.4	127 (28.2%)	24.0, 32.5	29 (15.4%)	9.51, 21.3
Violence against intimate partners :						
No violence	407 (70.9%)	66.6, 74,9	266 (59.2%)	53.6, 64.6	137 (73.7%)	66.1, 70.2
Physical violence only	125 (21.8%)	18.6, 25.3	124 (27.6%)	23.1, 32.6	37 (19.9%)	14.7, 26.3
Sexual violence only	23 (4.0%)	2.6, 6.1	19 (4.2%)	2.7, 6.7	5 (2.7%)	1.0, 7.2
Physical and sexual violence	19 (3.3%)	2.1, 5.1	40 (8.9%)	6.2, 12.6	7 (3.8%)	1.8, 7.6

Informed consent was signed for participation. Ethical approval was obtained from the University of Pretoria and University of the Witwatersrand Ethics Committees.

### Statistical analysis

All analyses took into account clustering and the multistage nature of the sample, with first a sample of 70 clusters being chosen and thereafter a sample of (up to) 20 men per cluster. Table 1 shows univariate analyses of variables in the dataset. Robust methods appropriate for the analysis of data from multistage sample surveys were used, with explanatory variables being summarized by the level of condom use (Table 2). For continuous variables, means and 95% confidence intervals are given, while for binary variables the number with the attribute, the percentage with the attribute and 95% confidence limits for the percentage are given.

A multinomial logistic regression model was fitted using the multilevel (xt) approach. Estimation of the parameters was carried out in STATA 10 using residual likelihood procedures. The model was built with inconsistent condom use as the reference variable versus non-condom use and consistent condom use. The variables listed in Table 2 were all considered as potential explanatory variables, all models contained a term for study stratum and were adjusted for age. In order to select variables in the model, variables were considered in groups namely socio-demographic variables, attitude variables, variables relating to the most recent or current relationship, and sexual practices and activities variables. For each group backward elimination was applied with a liberal nominal 20% significance level for exclusion, in order to identify a maximal subset of potential explanatory variables. A *P-*value of 0.05 was used for exclusion in the final model.

## Results

This paper analysed baseline interview data from 1219 young rural men aged 15–26 years who had ever had sexual intercourse. Table 1 shows that the sample mean age was 19.23 years (95% CI 19.06, 19.40). The majority of participants were poor with 36.5% (374) having very low SES scores, 17.6% (172) had mid scores and 46.9% (483) had were higher levels of SES. About half of the sample had done something to earn money in their lifetime. Risky sexual practices were common as half (52.1%) had had three or more sexual partners in the past year. Although two-thirds (66.8%) of men had ever used a condom, almost half of them had never used a condom in the past year, while amongst those who did 36.9% were inconsistent and 15.4% were consistent. On the composite violence against women variable, 23.7% reported only physical violence perpetration, 3.9% sexual violence and 5.5% physical or sexual violence. 18.2% had perpetrated raped of a non-partner. 98% of men were in the low or mid gender equity categories 52.0% (643) of men being the most gender inequitable, 45.9% (559) holding a middle position, and 2.1% (26) in the high equity category.

Table 2 shows bivariate associations between explanatory variables across three condom use categories. Inconsistent and consistent condom users were slightly older, and had higher socioeconomic status and had more money than never users. Consistent users showed progressive gender relations attitudes and less relationship control compared to other groups. Having 3 or more sexual partners was two times more likely amongst inconsistent users (82.0% inconsistent vs. 38.8% consistent and 33.3% never users). Inconsistent users (62.8%, 95% CI 52.5, 73.1) were also more likely to perpetrate violence against an intimate partner compared to never (39.7%, 95% CI 33.1, 46.4) and consistent users (36.6%, 95% CI 24.8, 48.3). 28.2% of inconsistent users reported perpetrating rape against a non-partner more often than their consistent (15.4%) or never (11.4%) condom use counterparts.

Table 3 presents the multinomial regression model of factors associated with inconsistent condom use in the past year (vs. consistent and never condom use). In comparison with the base group (inconsistent condom users), never users tended to be younger, poorer and less likely to have ever earned money (RRR 0.74, 95% CI 0.56, 0.97). They also held more conservative attitudes towards gender relations and were more controlling of partners (RRR 0.83, 95% CI 0.70, 0.97), and were significantly less likely to report have had 3 or more partners in the past year (RRR 0.13; 95% CI: 0.08, 0.16) and ever perpetrating rape against a non-partner (RRR 0.61; 95% CI: 0.41, 0.91) relative to inconsistent condom users. Consistent condom use reported significantly less risky practices as they were 86% less likely to have had three or more sexual partners in the past year (RRR 0.14; 95% CI 0.09, 0.22); and less likely to perpetrate physical violence towards an intimate sexual partner (RRR 0.66, 95% CI 0.45–0.98).

**Table 3:** Multinomial regression model of factors associated with consistent and no condom use versus inconsistent condom use among 1219 young men, adjusted for age

**Variables**	**Relative Risk Ratio**	**95% CI**	***P*-value**	**Relative Risk Ratio**	**95% CI**	***P*-value**
	**No condom use**			**Consistent condom use**		
Age in years	0.87	0.80, 0.95	0.002	1.00	0.89, 1.13	0.945
Higher socio-economic status	0.84	0.75, 0.94	0.003	Ns	ns	ns
Having gender relations attitudes & relationship control scale (high = liberal)	0.83	0.70, 0.97	0.019	Ns	ns	ns
Ever earned money	0.74	0.56, 0.97	0.031	Ns	ns	ns
Having 3 or more sexual partners in the past year	0.12	0.08, 0.16	<0.001	0.14	0.09, 0.21	<0.001
Ever perpetrated physical intimate partner violence	ns	Ns	ns	0.66	0.45, 0.98	0.041
Ever perpetrated non-partner rape	0.61	0.41, 0.91	0.016	ns	ns	ns

## Discussion

The paper examined the associations between patterns of condom use, and gender relations attitudes, and violence against intimate and non-partners and risky sexual practices. In the study setting, consistent condom use is an unconventional sexual practice with half of the men having never used condoms in the past year and inconsistent condom use being twice as likely as consistent use. Men who reported inconsistent condom use reported similar socio-economic background to consistent users but were more sexually risky and more violent compared to both never and consistent condom users.

The paper shows a tendency for clustering of young men’s characteristics around gender relations ideology, violent practices, sexual risk and socio-economic status by condom use group (see Table 2). We observed in the analysis an emergence of three male positions which distinguished the men from one another on the basis of gender attitudes and sexual/relationship practices. Never and consistent condom users were very similar with respect to violence perpetration and sexual risk taking, yet never condom users were markedly different from consistent users as they held very conservative attitudes towards gender relations and male control over female partners. Inconsistent users were very much more violent and sexually risky than the other groups, and held a middle position on gender attitudes. Whilst the gender attitudes of consistent users were not significantly different from inconsistent, this was a less violent and sexually risky masculinity. Given that the research was undertaken in what was mostly a deep rural area it is not surprising that there was little evidence of a very gender equitable masculinity as defined by Barker [35], but a recent rural South African study on male care work indicates that there is an emergence of a less domineering masculinity that is also conservative [17]. This analysis points to the need for nuance in understanding the non-linear relationship between violent and sexually risky men’s practices and attitudes towards gender equity and gender relations.

Inconsistent condom use is a risky sexual practice and places one at increased risk of HIV infection [36]. Similar tendencies to be violent are observed in another South African study where men who used condoms inconsistently were more likely to perpetrate physical/sexual intimate partner violence [37]. On its own, having many partners may also pose a difficulty in ensuring consistency of condom use with different partners, as authors have already shown the contradictions that may exists as to which partner condom use is more appropriate [14,15]. Thus, the current paper suggests that inconsistent condom use is part of a continuum of expressions of male heterosexuality which innately emphasize sexual conquest as a sign of a strong masculine image [38], and endorse the ideology pertaining to use of violence to control women [4]. This high reporting of such behaviours can be attributable to a heightened desire to embody a hegemonic masculinity described in Jewkes and Morrell [30]. Participants may not desire to be infected with HIV per se, but could be facing a composite challenge and contradictions that are posed by the notions of an ideal man as invincible, sexually virile and tough. Connell [28] refers to these contradictions as based on complicity with certain notions of manhood that are symbolic, familiar, manageable and also widely acceptable to a sector of men with whom one identifies. It appears that these young men who subscribe to such male violent and hypersexual ideals, including being lax about condom use, are indeed at greater risk of HIV infection and need to change their assumptions about who they are as men.

Never condom users portray a masculine position that is very conservative and yet less risky and violent, that is, very traditional but in some respects more ‘benign’ men. This suggests that such a masculinity may be in existence as mentioned in Jewkes and Morrell’s qualitative study: a female participant compared her two male partners, with one described as a very ‘traditional person’ who was very controlling but also allowed her some degree of freedom to socialise with her friends unlike her other partner who was expressly disapproving and controlling in the relationship [6]. The ‘never users’ were also much poorer than condom users. It’s hard to know whether they had fewer partners than inconsistent users because they lacked the money to entice women, or whether they simply did not aspire to be such men. Their lack of condom use could have been influenced by traditional ideas about sex, less exposure to more modern ideas and concerns about health and HIV risk, or it may be from assumptions of masculine invincibility [39]. Views about HIV invincibility may also underlie lower perceived HIV risk reported in other studies [40]. Never and consistent users were similar in terms of being benign towards women in their practices than inconsistent users, but differed in terms of gender attitudes. This may suggest that an important point for interventions may be to challenge traditional notions of masculinity by encouraging healthy sexual practices and men’s accessing of sexual and reproductive health services, thereby influencing a change in gender attitudes among men who are resistant to condom use.

Consistent users upheld more progressive gender relations attitudes but only in the bivariate analysis, and this is in direct opposite to never users who were similarly less sexually risky and less violent. The findings suggest that being more liberal on gender and relationships makes it permissible for men to intensify risk reduction strategies. The multinomial model showed that consistent condom users were less likely to be violent and had fewer sexual partners. Thus they appeared to represent another male position which is more benign, for example, being less violent towards an intimate partner and having fewer sexual partners are indicators of respectful and harmonious relationships [17]. A small proportion of men were gender equitable overall, however, the findings imply that gender equity is present in other practices such as observed in the consistent group thus supporting the notion that condom use is one of the male behaviours that could be considered when evaluating ideals of manhood, and being a consistent user does imply a progressive and healthy masculine position.

The findings draw into question an assumption that never condom users are the ‘riskiest’ group. The very high prevalence of risky sexual practices among inconsistent condom users indeed suggests that they may have been the ‘most vulnerable’ to HIV infection. Their higher levels of violent and sexually risk practices, as well as their relative conservatism, suggest that these men are an important target group for an intervention that seeks to change negative ideas about masculinity such as use of violence, having multiple concurrent partners and a precarious commitment to safer sex. Such interventions exist in South Africa, and these have shown success in engaging men at local and national levels, for example, Men as Partners [37]. However, there is uncertainty about the sustainability of these kinds of campaigns in rural settings but suggest that testing and subsequent wide up-scaling of such programmes in poor rural communities of South Africa can have far-reaching effects in curbing the incidence of HIV over time. The Brother’s for Life initiative mainly targets men over the age of 30 years on collectively addressing risky sexual behavior, gender based violence and promoting HIV prevention and male health seeking and participation through multimedia, and presents a model that could be adapted for a younger age group of men.

### Limitations

The sample was largely homogeneous in terms of demographic and socioeconomic factors. Since this data analysed for this paper is cross-sectional, we cannot draw any causal inferences from our findings, but can point to observed clustering of practices which has been discussed elsewhere [30]. The study setting and participants were not randomly selected which limits generalisability. This may not affect the association between variables and associations found have often confirmed those of other authors, and therefore we have confidence that the findings of this study have validity. The consistent condom group is much smaller than the other groups and this will have widened confidence intervals, but this does not cast doubt on the reliability of the findings as the standard and rigorous statistical measures were used, to establish and test the associations of variables with condom user group. A longitudinal study to investigate the role of masculine gender ideologies on men’s condom use and other sexually risky practices may be valuable. The study relied on self-reported behaviour, which is prone to desirability bias. This may have been minimised by using just a few interviewers (56% of the interviews were done by 2 men) who received intensive initial and on-going training and support and were similar of age group, sex and background to the study sample [32].

## Conclusions

Our findings appear to support the hypothesis that gender and relationship constructs significantly determine condom use patterns of rural young men who participated in a cross-sectional South Africa study. Though this paper focuses on condom practices, there is ample evidence to corroborate views that HIV risk is driven by risky sex and violent and illiberal gender relations towards women. As with the ecological model [11], there are multiple levels of influence on condom behavior as the confluence of conservative gender attitudes, elevated use of violence against women and sexual risk taking shown here has rendered inconsistent condom users the more risky than non-users, and presented them as possessing one of the ‘negative’ configurations of hegemonic masculinity in the study setting. Changing non- and inconsistent condom use to consistent use is not only possible amongst youth, but also an important step in their efforts to prevent HIV infection and should be optimally promoted in HIV risk reduction interventions going beyond the ABC messaging and condom demonstrations. Using a condom consistently should be promoted as a positive, progressive and healthy attribute of successful masculinity, along with promotion of gender equity and male participation in sexual and reproductive health. Programmes that are targeted at engaging men in HIV prevention and building gender equity, namely Men as Partners [37] and Stepping Stones [33], have demonstrated positive behaviour change effects and were scientifically tested within the South Africa context, yet the determination to roll these programmes out at a national scale seems to be lacking.

## Competing interests

The study sponsors have had no involvement in the study design, analysis or interpretation of the findings or writing of the paper.

## Non-financial competing interests

I hereby declare that there are no non-financial competing interests.

## Authors’ contributions

NJS came up with research question for her PhD studies, drafted the manuscript, contributed to the study design, data collection, data analysis and writing of this article.

RJ developed the randomised control study from which the data of this manuscript has been selected, approved the research question, data analysis and writing of the manuscript as the PhD supervisor, and approves the manuscript to be published.

MN contributed to the study design, data collection, and reviewed the manuscript and approves the submission of the manuscript for publication.

KD contributed to the study design, data collection, data analysis and reviewed the manuscript, and approves the submission of the manuscript for publication.

All authors read and approved the final manuscript.

## Pre-publication history

The pre-publication history for this paper can be accessed here:

http://www.biomedcentral.com/1471-2458/12/462/prepub

## References

[B1] Harrison A Cleland J Gouws E Frohlich J Early sexual debut among young men in rural South Africa: heightened vulnerability to sexual risk? Sex Transm Infect 2005 81 259 261 10.1136/sti.2004.011486 15923298 PMC1744981

[B2] Pettifor AE Rees HV Steffenson A Hlongwa-Madikizela L MacPhail C Vermaak K Kleinschmidt I HIV and Sexual Behaviour Among Young South Africans : A National Survey of 15–24 year olds Book HIV and Sexual Behaviour Among Young South Africans : A National Survey of 15–24 year olds. (Editor ed.^eds.) 2004 Reproductive Health Research Unit, University of Witwatersrand, http://www.lovelife.org.za/research/studies.php

[B3] Moyo W Levandowski BA MacPhail C Rees H Pettifor A Consistent condom use in South African youth’s most recent sexual relationships AIDS Behav 2008 12 431 440 10.1007/s10461-007-9343-3 18228125

[B4] Harrison A O’Sullivan LF Hoffman S Dolezal C Morrell R Gender Role and Relationship Norms among Young Adults in South Africa: Measuring the Context of Masculinity and HIV Risk J Urban Health 2006 83 709 722 10.1007/s11524-006-9077-y 16758334 PMC2430491

[B5] Jewkes RK Levin JB Penn-Kekana LA Gender inequalities, intimate partner violence and HIV preventive practices: findings of a South African cross-sectional study Soc Sci Med 2003 56 125 134 10.1016/S0277-9536(02)00012-6 12435556

[B6] Jewkes R Morrell R Sexuality and the limits of agency among South African teenage women: Theorising femininities and their connections to HIV risk practises Soc Sci Med 2012 74 1729 1737 10.1016/j.socscimed.2011.05.020 21696874 PMC3217103

[B7] Pettifor AE Measham DM Rees HV Padian NS Sexual power and HIV risk, South Africa Emerg Infect Dis 2004 10 1996 2004 10.3201/eid1011.040252 15550214 PMC3328992

[B8] Noar S Webb E Van Stee S Feist-Price S Crosby R Willoughby J Troutman A Sexual Partnerships, Risk Behaviors, and Condom Use Among Low-Income Heterosexual African Americans: A Qualitative Study Arch Sex Behav 2011 1 12 https://springerlink3.metapress.com/content/3gng11072127h972/resource-secured/?target=fulltext.html&sid=goz10yoi4orr4u35fomzpox4&sh=www.springerlink.com 22194089 10.1007/s10508-011-9890-6 PMC3352984

[B9] Dunkle KL Jewkes RK Brown HC Gray GE McIntryre JA Harlow SD Gender-based violence, relationship power, and risk of HIV infection in women attending antenatal clinics in South Africa Lancet 2004 363 1415 1421 10.1016/S0140-6736(04)16098-4 15121402

[B10] Jewkes R Dunkle K Nduna M Shai N Intimate partner violence, relationship power inequity, and incidence of HIV infection in young women in South Africa: a cohort study Lancet 2010 376 41 48 10.1016/S0140-6736(10)60548-X 20557928

[B11] Sallis JF Owen N Fisher EB Glanz K, Rimer BK, Viswanath K Ecological Models of Health Behavior Health Behavior and Health Education: Theory, Research and Practice 2008 4 San Francisco, CA: Jossey-Bass, A Wiley Imprint

[B12] Sales JM Lang DL DiClemente RJ Latham TP Wingood GM Hardin JW Rose ES The mediating role of partner communication frequency on condom use among African American adolescent females participating in an HIV prevention intervention Heal Psychol 2012 31 63 69 10.1037/a0025073 PMC3242911 21843001

[B13] Varga CA Sexual decision-making and negotiation in the midst of AIDS: youth in KwaZulu-Natal, South Africa Health Transit Rev 1997 7 45 67

[B14] Ragnarsson A Townsend L Thorson A Chopra M Ekstrom AM Social networks and concurrent sexual relationships–a qualitative study among men in an urban South African community AIDS Care 2009 21 1253 1258 10.1080/09540120902814361 20024701

[B15] Kenyon C Boulle A Badri M Asselman V “I don’t use a condom (with my regular partner) because I know that I’m faithful, but with everyone else I do”: The cultural and socioeconomic determinants of sexual partner concurrency in young South Africans J Soc Aspects HIV/AIDS 2010 7 35 43 10.1080/17290376.2010.9724967 21409303

[B16] Santana MC Raj A Decker MR La Marche A Silverman JG Masculine gender roles associated with increased sexual risk and intimate partner violence perpetration among young adult men J Urban Health 2006 83 575 585 10.1007/s11524-006-9061-6 16845496 PMC2430489

[B17] Jama Shai N Jewkes R Levin J Dunkle K Nduna M Factors associated with consistent condom use among rural young women in South Africa AIDS Care 2010 22 1379 1385 10.1080/09540121003758465 20730637

[B18] Pettifor AE Rees HV Kleinschmidt I Steffenson EA MacPhail C Hlongwa-Madikizela L Vermaak K Padian NS Young people’s sexual health in South Africa: HIV prevalence and sexual behaviors from a nationally representative household survey AIDS 2005 19 1525 10.1097/01.aids.0000183129.16830.06 16135907

[B19] Jewkes R Sikweyiya Y Morrell R Dunkle K Understanding men’s health and use of violence: interface of rape and HIV in South Africa. Technical Report 2009 Pretoria: Medical Research Council http://www.mrc.ac.za/gender/projects.htm

[B20] Jewkes R Morrell R Sexuality and the limits of agency among South African teenage women: Theorising femininities and their connections to HIV risk practises Soc Sci Med 2012 74 11 1729 37 Epub 2011 May 31 10.1016/j.socscimed.2011.05.020 21696874 PMC3217103

[B21] Jewkes R Sikweyiya Y Morrell R Dunkle K The Relationship between Intimate Partner Violence, Rape and HIV amongst South African Men: A Cross-Sectional Study PLoS One 2011 6 e24256 10.1371/journal.pone.0024256 21935392 PMC3173408

[B22] Silverman JG Decker MR Saggurti N Balaiah D Raj A Intimate partner violence and HIV infection among married Indian women Jama 2008 300 703 710 10.1001/jama.300.6.703 18698068

[B23] Dunkle KL Jewkes RK Nduna M Levin J Jama N Khuzwayo N Koss MP Duvvury N Perpetration of partner violence and HIV risk behaviour among young men in the rural Eastern Cape, South Africa AIDS 2006 20 2107 2114 2110.1097/2101.aids.0000247582.0000200826.0000247552 10.1097/01.aids.0000247582.00826.52 17053357

[B24] Townsend L Rosenthal SR Parry CD Zembe Y Mathews C Flisher AJ Associations between alcohol misuse and risks for HIV infection among men who have multiple female sexual partners in Cape Town, South Africa AIDS Care 2010 22 1544 1554 10.1080/09540121.2010.482128 20824551

[B25] Silverman JG Decker MR Kapur NA Gupta J Raj A Violence against wives, sexual risk and sexually transmitted infection among Bangladeshi men Sex Transm Infect 2007 83 211 215 17301104 10.1136/sti.2006.023366 PMC2659096

[B26] Jewkes R Dunkle K Koss MP Levin JB Nduna M Jama N Sikweyiya Y Rape perpetration by young, rural South African men: Prevalence, patterns and risk factors Soc Sci Med 2006 63 2949 2961 10.1016/j.socscimed.2006.07.027 16962222

[B27] Stephenson R Community-Level Gender Equity and Extramarital Sexual Risk-Taking AmongMarriedMen in Eight African Countries Int Perspect Sex Reprod Heal 2010 36 178 188 10.1363/3617810 PMC3960719 21245024

[B28] Connell RW Gender and power: society, the person, and sexual politics 1987 Stanford, California, USA: Stanford University Press annotated, reprint

[B29] Connell RW Hegemonic Masculinity: Rethinking the Concept Gend Soc 2005 19 829 859 10.1177/0891243205278639

[B30] Jewkes R Morrell R Gender and sexuality: emerging perspectives from the heterosexual epidemic in South Africa and implications for HIV risk and prevention J Int AIDS Soc 2010 13 1 11 10.1186/1758-2652-13-1 20181124 PMC2828994

[B31] Jewkes R Nduna M Jama PN Stepping Stones, South African Adaptation Book Stepping Stones, South African Adaptation. (Editor ed.^eds.) 2002 2 South Africa: Medical Research Council

[B32] Jewkes R Nduna M Levin J Jama N Dunkle K Khuzwayo N Koss M Puren A Wood K Duvvury N A cluster randomized-controlled trial to determine the effectiveness of Stepping Stones in preventing HIV infections and promoting safer sexual behaviour amongst youth in the rural Eastern Cape, South Africa: trial design, methods and baseline findings Trop Med Int Health 2006 11 3 16 10.1111/j.1365-3156.2005.01530.x 16398750

[B33] Jewkes R Nduna M Levin J Jama N Dunkle K Puren A Duvvury N Impact of Stepping Stones on incidence of HIV and HSV-2 and sexual behaviour in rural South Africa: cluster randomised controlled trial British Med J 2008 337 a506 10.1136/bmj.a506 PMC2505093 18687720

[B34] WHO Multi-Country Study on Women’s Health and Domestic Violence: Core Questionnaire and WHO Instrument 2000 Version 9. Geneva, Switzerland: World Health Organisation

[B35] Barker G Gender equitable boys in a gender inequitable world: reflections from qualitative research and programme development in Rio de Janeiro Sex Relationsh Ther 2000 15 263 282 10.1080/14681990050109854

[B36] Weller SC Davis-Beaty K Condom effectiveness in reducing heterosexual HIV transmission Cochrane Database Syst Rev 2002 10.1002/14651858.CD003255 PMC8407100 11869658

[B37] Peacock D Levack A The Men as Partners Program in South Africa: Reaching Men to End Gender-Based Violence and Promote Sexual and Reproductive Health Int J Men’s Health 2004 3 173 188 10.3149/jmh.0303.173

[B38] Varga CA How Gender Roles Influence Sexual and Reproductive Health Among South African Adolescents Stud Fam Plan 2003 34 160 172 10.1111/j.1728-4465.2003.00160.x 14558319

[B39] Courtenay WH Constructions of masculinity and their influence on men’s well-being: a theory of gender and health Soc Sci Med 2000 50 1385 1401 10.1016/S0277-9536(99)00390-1 10741575

[B40] MacPhail C Campbell C ‘I think condoms are good but, aai, I hate those things’: condom use among adolescents and young people in a Southern African township Soc sci med 2001 52 1613 1627 10.1016/S0277-9536(00)00272-0 11327136

